# Interaction of Chandipura Virus N and P Proteins: Identification of Two Mutually Exclusive Domains of N Involved in Interaction with P

**DOI:** 10.1371/journal.pone.0034623

**Published:** 2012-04-02

**Authors:** Arindam Mondal, Arunava Roy, Sandipto Sarkar, Jishnu Mukherjee, Tridib Ganguly, Dhrubajyoti Chattopadhyay

**Affiliations:** 1 Department of Biotechnology and Dr. B. C. Guha Centre for Genetic Engineering and Biotechnology, University of Calcutta, Kolkata, West Bengal, India; 2 Department of Biological Sciences, IISER, Kolkata, West Bengal, India; International Centre for Genetic Engineering and Biotechnology, Italy

## Abstract

The nucleocapsid protein (N) and the phosphoprotein (P) of nonsegmented negative-strand (NNS) RNA viruses interact with each other to accomplish two crucial events necessary for the viral replication cycle. First, the P protein binds to the aggregation prone nascent N molecules maintaining them in a soluble monomeric (N^0^) form (N^0^-P complex). It is this form that is competent for specific encapsidation of the viral genome. Second, the P protein binds to oligomeric N in the nucleoprotein complex (N-RNA-P complex), and thereby facilitates the recruitment of the viral polymerase (L) onto its template. All previous attempts to study these complexes relied on co-expression of the two proteins in diverse systems. In this study, we have characterised these different modes of N-P interaction in detail and for the first time have been able to reconstitute these complexes individually *in vitro* in the chandipura virus (CHPV), a human pathogenic NNS RNA virus. Using a battery of truncated mutants of the N protein, we have been able to identify two mutually exclusive domains of N involved in differential interaction with the P protein. An unique N-terminal binding site, comprising of amino acids (aa) 1–180 form the N^0^-P interacting region, whereas, C-terminal residues spanning aa 320–390 is instrumental in N-RNA-P interactions. Significantly, the e*x-vivo* data also supports these observations. Based on these results, we suggest that the P protein acts as N-specific chaperone and thereby partially masking the N-N self-association region, which leads to the specific recognition of viral genome RNA by N^0^.

## Introduction

Chandipura virus (CHPV) is a prototype member of the family *Rhabdoviridae* in the order *Mononegavirales*, which also includes vesicular stomatitis virus (VSV) and rabies virus (RAV). This virus belongs to the broader group of negative-strand RNA viruses (NSRVs), which includes many pathogenically significant viruses, like avian influenza, measles, and Ebola. CHPV has repetitively caused severe outbreaks of encephalitis in parts of India [1]–[6] and has recently been classified as an emerging human pathogen in the Indian subcontinent [4], [7]. Like other members of the Rhabdovirus family, its single-stranded, negative-sense RNA genome is encapsidated with the nucleocapsid protein (N) into a helical nucleocapsid (NC) structure which together with the viral RNA dependent RNA polymerase (RdRp) components, the large protein (L) and the phosphoprotein (P), is packaged into the virion particle. This genome RNA enwrapped within the nucleocapsid in association with the viral RdRp forms the Ribonucleoprotein particle (RNP), a self-sufficient entity for infection [8].

Encapsidation of the genome RNA by N protein is not only essential for protecting the viral genome from RNase action, but, is also believed to play a major role in the switching of the viral transcription to replication mode [9]. A unique characteristic feature of all nonsegmented negative-strand RNA viruses (NNSRVs) is that the active template for RNA polymerization reactions is the encapsidated genome RNA, and never the naked RNA. Also, during replication, the nascent RNA is encapsidated concomitantly to its synthesis so that it can sustain subsequent rounds of replication, or be packaged within the virion particle. This is supported by the observation that continuous synthesis of N protein and its stoichiometric availability is indispensible during viral replication [10]–[13]. One fundamental requirement for the N protein is that it must specifically encapsidate the viral genomic RNA and not non-specific cellular RNAs. However, the rhabdoviral N protein alone is incapable of performing this task. According to numerous studies, N protein when expressed alone forms large insoluble aggregates [14]–[16] and binds to short cellular RNAs non-specifically [17]–[21]. Conversely, when co-expressed with the P protein, a major fraction of the N protein is rendered soluble and free of non-specific RNA. P was reported to form complex with monomer N (N^0^-P) [22]–[25] thereby inhibiting N-N self association [16], [26]–[28] and also imparting specificity towards viral RNA sequence [26], [28], [29]. Therefore, interaction with the P protein is an imperative requirement for the maintenance of encapsidation competent N protein. On the other hand, P protein also interacts with the mature nucleocapsid in order to recruit the L polymerase onto its template, as the L protein cannot bind to the N-RNA template by itself [30], [31]. This interaction has been studied in considerable details by analysing the crystal structure of VSV nucleocapsid like particles (NLP) in association with the C-terminal domain of P (P_CTD_). It showed that decameric structures of N enwrapping a 90 nt RNA which remains associated with 5 molecules of P protein (N∶P molar ratio of 2∶1) [18], [32]. The C-terminal domains of both N and P proteins were found to participate in such N-RNA-P complex formation [18], [32].

However, for the monomer N-P complex (N^0^-P), the interacting domains of N, and the nature of the interaction at large, remains uncharacterised. One of the major obstacles for such biochemical and structural studies has been the unavailability of soluble N^0^ protein. However, recently Leyrat et al. has reported the structure of a N-terminal 21 aa deleted version of the VSV N protein with the first 60 aa of VSV P (N_Δ21_^0^-P_60_) [33]. More significant progress has been made in regard to the N^0^ interacting domains of the P protein. Chen et. al. has shown that for VSV, N-terminal 11–30 amino acids of P protein are essential in keeping N in soluble form (N^0^-P complex formation) [14]. This N^0^-binding region of VSV P was shown to be globally disordered and encompasses the transient α-helices [34]. Similar observations have been made for Rabies, Sendai and HPIV3 viruses, where N-terminal 40 aa of P protein has been implicated for N^0^-P interactions [35]–[37]. These studies pointed towards the fact that P protein has independent domains for interaction with monomer and oligomeric N protein. The presence of two separate N binding regions in P, strongly suggests that the N protein might also utilize two separate P binding domains in its different oligomeric states, i.e. N^0^ and N-RNA.

In this study, we chose to characterise the P binding region(s) of CHPV N. Recently, we have delineated the self-association and RNA binding domains of CHPV N [21]. It was found that the N-terminal 47 aa together with residues 180–264 were important for proper nucleocapsid like structure formation, while the C-terminal domain was found dispensable for the same. Interestingly, it was also found that the RNA recognition event was a function of the oligomerization status of the protein. In brief, the C-terminal 102 aa (residues 320–422) was found to be important for the specific recognition of the leader RNA sequence of CHPV, and this function is active only under monomeric conditions of the protein. Upon oligomerization, the RNA recognition specificity is lost and the N-terminal domain was found to mediate the non-specific and progressive enwrapping of the genome RNA. In this current work, we have characterized the different modes of CHPV N-P interactions in *ex-vivo* and in *in vitro* systems using deletion mutagenesis. Our results indicate the presence of two mutually exclusive P interacting domains in CHPV N. Consistent to previous observations in related viruses, a C-terminal P binding region has been observed that is functional only under oligomeric condition. In addition, a previously unknown N-terminal region of N has been identified that binds to P only in its monomeric form (N^0^-P). This helps sheds light upon the intricate molecular machinery that controls encapsidation in CHPV and other rhabdoviruses at large. We have also suggested a model that helps to explain the molecular basis of the N specific chaperone like activity of P protein, and subsequent specific encapsidation of the viral RNA by N.

## Results

### Study of the interaction between CHPV N and P proteins in Vero-76 cells

**Figure 1:**
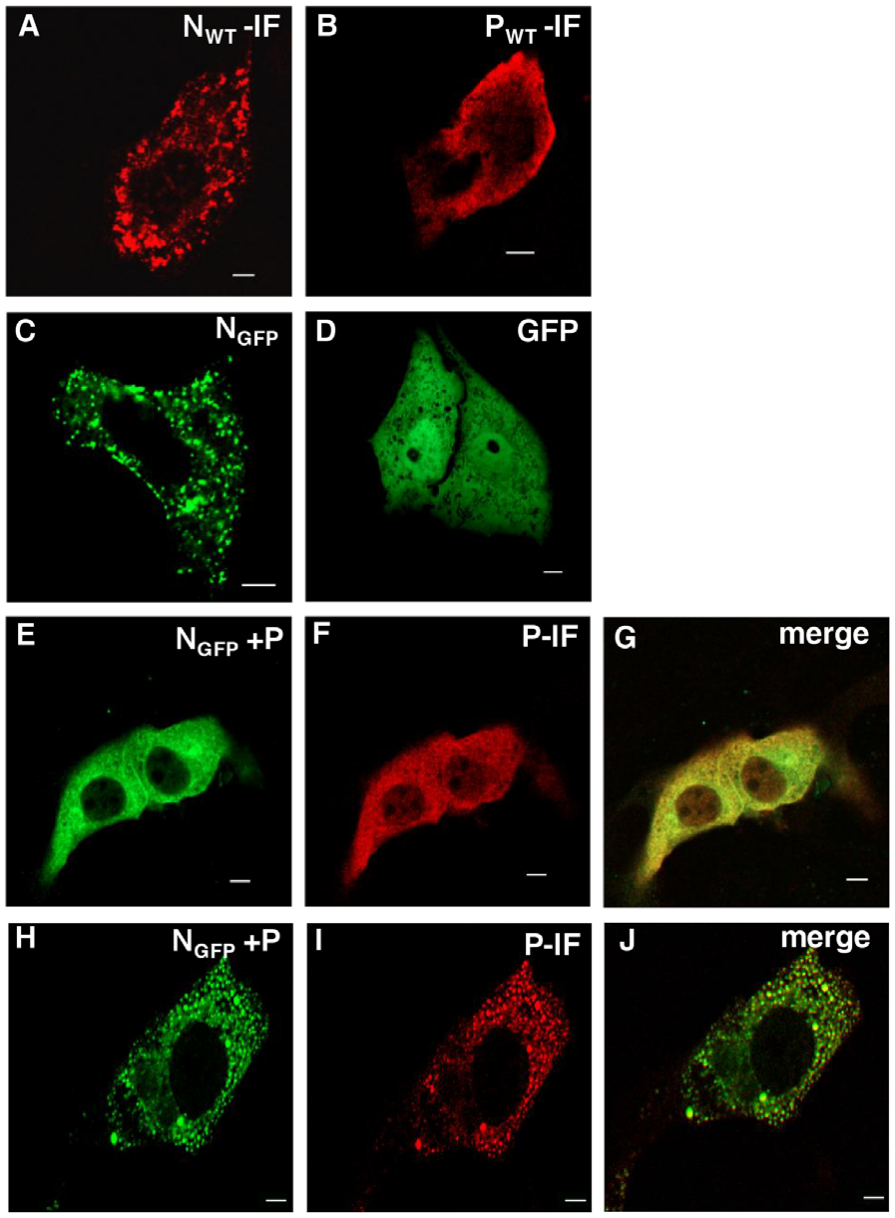
CHPV N and P proteins interact differentially in transfected cells depending on their stoichiometric availabilities. (A) Vero-76 cells were transfected with 2 µg pCDNA 3.1 (+) N and immunofluorescence performed with N-Ab, 24 hours post transfection. N exhibits a punctate distribution in the cytoplasm. (B) Immunofluorescence of Vero-76 cells transfected with pCDNA 3.1 (+) P, with P-Ab. P exhibits a smooth distribution in the cytoplasm. (C) GFP fluorescence of Vero-76 cells transfected with pEGFP-C1 N. GFP-tagged N maintains its punctuated distribution. (D) GFP fluorescence of Vero-76 cells transfected with pEGFP-C1 vector alone. GFP alone shows characteristic smooth fluorescence throughout the cell. (E to G) Vero-76 cells co-transfected with pEGFP-C1 N and pCDNA 3.1 (+) P in a 1∶1 ratio. P was detected by immunofluorescence (F). Colocalization of GFP-N with P is shown in the merged image (G). Co-expression with P redistributes the otherwise punctuated N into a more homogenous fluorescence. (H to J) Co-transfection in a 1∶0.5 ratio. The lower abundance of P is insufficient to homogenise the punctuated distribution of N (H). Immunofluorescence against P reveals colocalization of P with oligomeric forms of N (I and J). All data were captured on a laser scanning confocal microscope (Carl Zeiss). All Immunofluorescence were performed with anti-rabbit TRITC conjugated secondary antibody. 2 µg of DNA was used for all transfection, except for H, I and J where 1 µg of pCDNA 3.1 (+) P was used. The bar represents 5 µm.

To monitor the interaction of CHPV N and P proteins in living cells, in isolation of other viral proteins, the two proteins were either expressed individually or co-expressed in different molar ratios in Vero-76 cell line. Immunofluorescence against untagged N (pCDNA3.1(+) N) and P protein (pCDNA3.1(+) P) reveals that, when expressed alone, N protein exhibits a punctate distribution, while P protein demonstrated a homogeneous distribution, throughout the cytoplasm (Figure 1A and B). Transfection of an N-terminal EGFP tagged N construct resulted in a similar punctate distribution. EGFP alone has a characteristic homogenous distribution throughout the cell (Figure 1C and D); thus implicating that EGFP fusion does not have any effect upon the intracellular distribution of the protein and it is N, that is responsible for conferring such a punctate distribution of the fusion protein. Similar distribution of EGFP tagged CHPV N protein has also been reported earlier [16] and could be attributed to the self-association character of the protein [21], [29].

Co-transfection of the plasmids encoding EGFP-N and untagged P proteins in a 1∶1 ratio resulted in redistribution of the punctate structures of N into a complete homogenous distribution (Figure 1E). Among cells exhibiting GFP fluorescence, about 90% showed such change in the distribution pattern of N. Immunofluorescence against P (red), confirmed co-expression of N and P proteins in these cells and its co-localization with N (Figure 1F and G). The remaining 10% cells, was found to be lacking in the expression of P, and therefore showed characteristic punctate structures of N (Figure S1). It is thus evident that the P protein can impart chaperone-like activity in the intercellular milieu for the solubilisation of the otherwise punctate N protein. On the contrary, co-transfection of plasmids encoding EGFP-N and P proteins in 1∶0.5 ratio resulted in an entirely different observation. In this case, P failed to homogenize the punctate distribution of N, and interestingly, the P specific fluorescence (red) was found to completely co-localized with the aggregated structures of N (Figure 1H to J). It appears that, when co-expressed in 1∶0.5 ratio, the stoichiometric availability of P is insufficient for it to exert its chaperone like activity upon N. However, co-localization of P with the punctate structures of N under this condition confirms the ability of P to interact with aggregated N.

**Figure 2:**
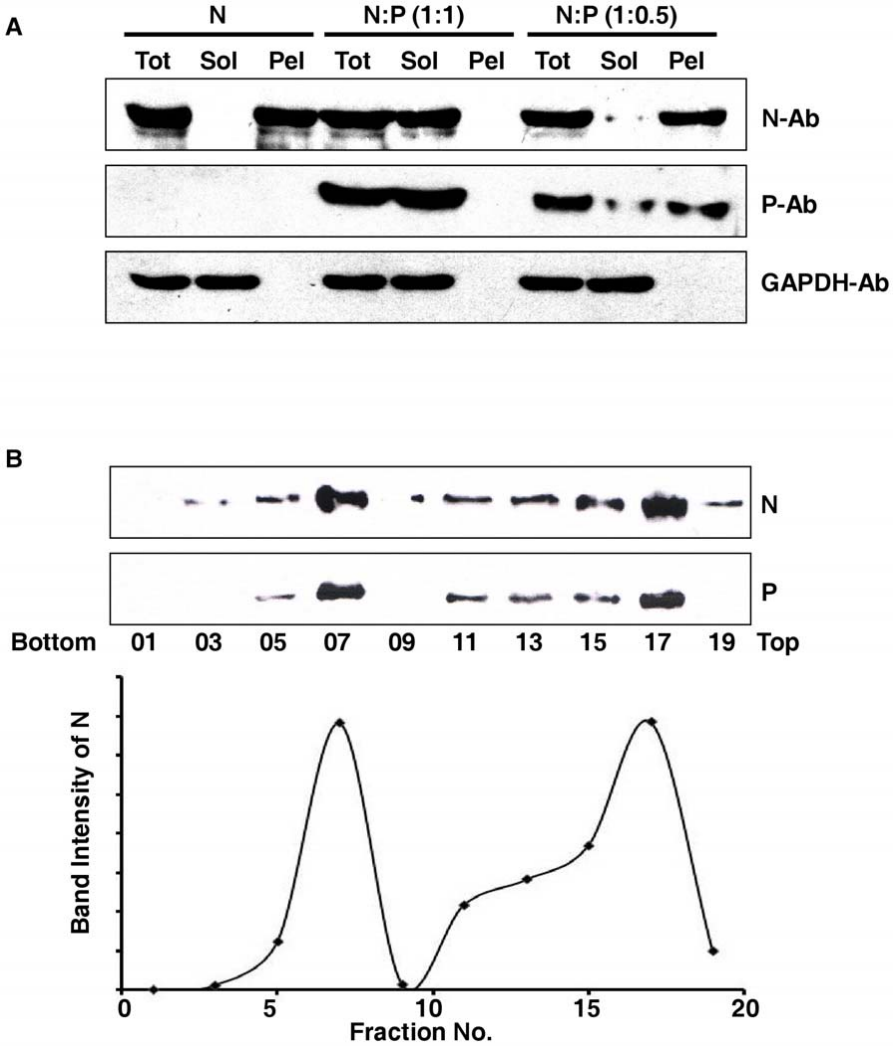
Soluble-insoluble fractionation and sucrose density gradient centrifugation. Stoichiometry of N and P ratio is important for the N specific chaperone activity of P. (A) Total (Tot), Soluble (Sol) and Insoluble (Pel) fractionation of Vero-76 cells transfected with different ratios of pEGFP-C1 N and pCDNA 3.1 (+) P at 24 hours post-transfection. N and P proteins were detected by immunoblotting with N and P Ab respectively. It is evident that a 1∶0.5 N-P ratio is incapable of solubilising the otherwise insoluble N; however, a 1∶1 ratio can do so. GAPDH was used as a loading control. (B) Oligomerization status of soluble N. Sucrose density gradient centrifugation of the soluble fraction of cells transfected with 1∶1 ratio of GFP-N and P constructs. Fractions were collected from the bottom of the tube, and alternative fractions were immunobloted with N and P Abs. The curve shows the band intensities representing distribution of GFP-N and P against the fraction number. While majority of the soluble fraction of N is found in the monomeric form (fraction 17), a substantial amount is also found in fraction 7, indicating decameric forms. P is found to interact with both the populations of N. However, other stoichiometries of homo-oligomerization cannot be ruled out (fractions 13 through 17).

To further validate this data, cells expressing either EGFP-N alone or EGFP-N and P together in different ratios were lysed and centrifuged at 13,000 rpm. Supernatant and pellet fractions were analyzed for N and P using specific antibodies (Figure 2A). As expected, co-expression in 1∶1 ratio resulted in solubilisation of a major fraction of the otherwise insoluble N, re-establishing the chaperone like activity of P [16], [27]. However, when co-transfected in 1∶0.5 ratio, N was majorly found in the pellet fraction. This confirms that at 1∶0.5 ratio P fails to exert its chaperone like activity upon N. In addition, P protein, which is generally soluble, was found almost entirely in the pellet fraction when co-transfected with N in 1∶0.5 ratio. This could be a result of its interaction with aggregated insoluble N. The supernatant fraction obtained from the co-expression of N-P in 1∶1 ratio, were analyzed by centrifugation through a 10–60% sucrose density gradient to determine the oligomerization status of N in this soluble preparation. Fractions were collected from the bottom of the gradient and blotted against N and P (Figure 2B). Interestingly, there are two major populations of N widely separated from one another. A significant population was found to penetrate up to the 7^th^ fraction; showing considerably higher sedimentation value than decameric N (10^th^ fraction, as shown previously in [21]). Immunoblotting with P antibody confirmed the association of P with this population of N. This accounts for its higher sedimentation value than decameric N (Figure 2B, lower panel). The second major population of N, although remained associated with P, was found to have a much lower sedimentation velocity (penetrated up to the 17^th^ fraction only). It seems that this population represents a low molecular weight complex, consisting of single subunits of N^0^-P proteins, as depicted by sedimentation values. Although, it is difficult to determine the exact stoichiometry of N to P, this experiment clearly shows the formation of two distinct complexes among N and P when co-expressed. In addition, presence of RNA in the two complexes was tested by measuring their A_280 nm_/A_260 nm_ ratio. Higher RNA content should be reflected by a lower A_280 nm_/A_260 nm_ ratio compared to fractions with lesser or no RNA content. The larger complex (7^th^ fraction) corresponding to decameric N-P populations, exhibited a much lower A_280 nm_/A_260 nm_ ratio compared to the 17^th^ fraction, which corresponds to N^0^-P complexes (data not shown). This confirms that the larger complex is an N-RNA-P complex, where decameric N have encapsidated cellular RNAs non-specifically. The smaller N^0^-P complex seems to be devoid of any cellular RNA.

### N forms distinct complexes with P *in vitro*

**Figure 3:**
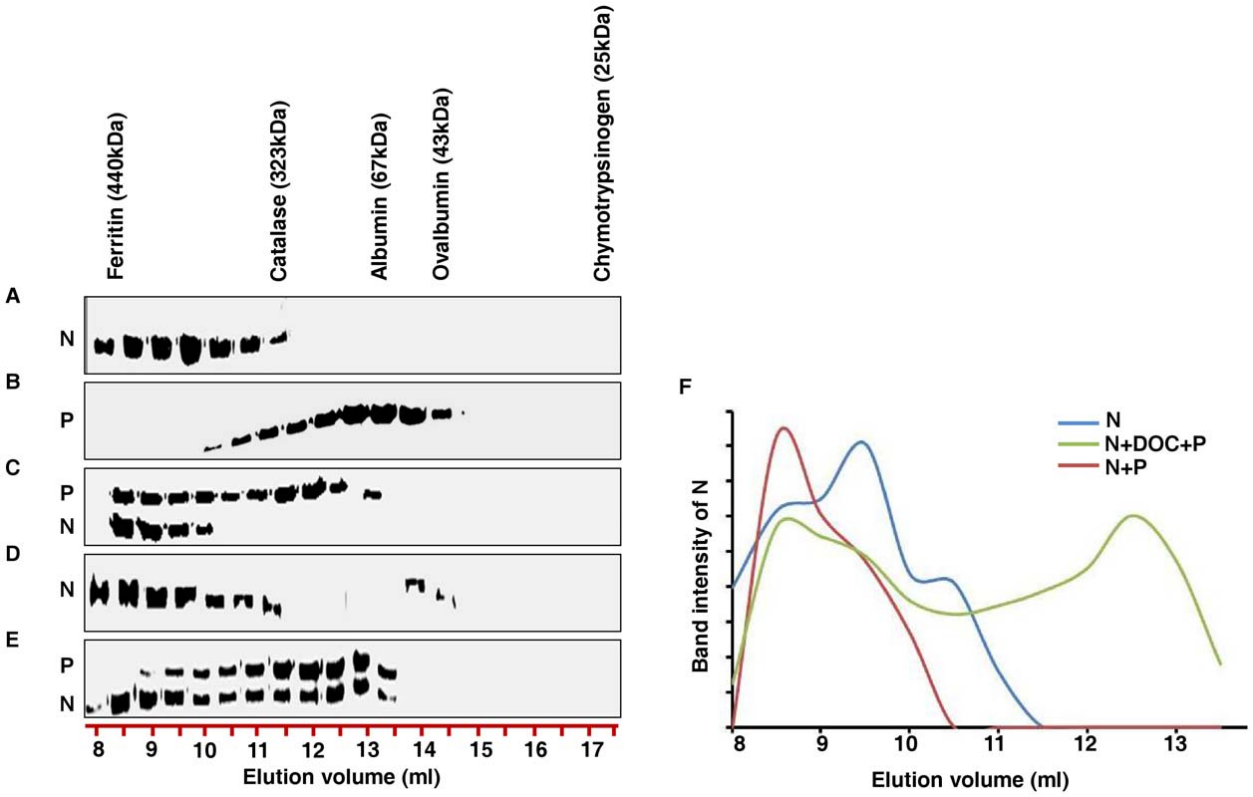
N forms distinct complexes with P *in vitro*. Size exclusion chromatography of bacterially expressed purified N and P proteins, visualised by Coomasie brilliant blue staining. (A) N alone shows higher oligomeric distribution, suggesting decameric species. (B) P alone shows characteristic dimeric forms. (C) N and P incubated together at 4°C for 30 minutes. Interaction between N (oligomer) and P is evident, as they co-elute just after the void volume fraction. (D) N treated with 1% DOC for 30 minutes and subsequently dialysed to remove DOC. Though DOC treatment dissociated the oligomeric N into monomeric forms, removal of DOC by dialysis allows them to re-oligomerize. (E) Similar to D, except for the fact that DOC was removed in the presence of equimolar concentration of P protein. Presence of P protein during DOC removal retains the monomeric population of N, and subsequently, monomer N-P complexes elute at around 12 ml. Elution profiles of gel filtration standard are shown for molecular weight estimation. (F) Plot representing densitometric scans of N bands with respect to elution volume in ml.

Next, a cell free assay system was employed to further characterize the different N-P interactions and the detail stoichiometry involved in it. To this end, we have performed size-exclusion chromatography through Superdex-200 column using bacterially expressed, purified N and P proteins [27], [38]. In tune with previous reports [21], [29], N protein showed characteristic oligomerization pattern in size-exclusion chromatography, eluting at around 9.5 ml, between ferritin and catalase (Figure 3A). We have previously reported that this population of N represents ring shaped nucleocapsid like particle under transmission electron microscopy [21]. P eluted at 13.25 ml (Figure 3B), suggesting that a major fraction of the protein remains in dimer form, thereby validating the concentration dependent dimerization property of this protein [39]. Interestingly, prior incubation of N and P together at 4°C for 30 minutes resulted in formation of a high molecular weight complex which eluted at 8.5 ml (Figure 3C). The exact molecular weight of the complex was difficult to determine as the elution volume is close to the void volume of the column. The molar ratio of N to P in this species was determined by densitometric analysis to be 2∶1. Therefore, it can be inferred that the P protein interacts with the ring shaped nucleocapsid like particles to form this high molecular weight complex.

Treatment of CHPV N with Sodium Deoxycholate (DOC) results in the dissociation of the N oligomers into monomers [29]. This phenomenon has been used to confirm the P interacting ability of monomer N as previously indicated by the density gradient results (Figure 2B). Bacterially expressed, purified N was treated with 1% DOC for 30 minutes at 4°C and subsequently DOC was removed by dialysis either in the absence or presence of P protein. The resulting complex was subjected to gel-filtration chromatography (Figure 3D and E respectively). In absence of P, removal of DOC resulted in the restoration of oligomeric status of N protein as evident from the identical gel-filtration profile encountered without DOC treatment (compare Figure 3A and D). However, presence of P during DOC removal resulted in a substantial change in the gel-filtration profile of N (compare 3A with E and Figure 3F). The result shows that, a significant fraction of N interacts with the P protein to form a series of low molecular weight complexes at the expense of the high molecular weight complex mentioned above (compare Figure 3E and C). Majority of these complexes eluted between 12–13 ml (Figure 3F) in a range between 100-80 kDa. Interaction of a single subunit of N with either monomer or dimer P could account for such low molecular weight complexes. This possibility was also supported by the densitometric analysis of respective bands. While for the 12^th^ ml fraction (Figure 3E), the estimated N to P ratio was 1∶2, for the 12.5–13 ml fractions it was 1∶1. Clearly, P exerts chaperone like activity upon N, thereby inhibiting its self-association and thus resulting in its slower elution in size-exclusion chromatography. Till date, such N-P complexes have been encountered only upon co-expression of both the proteins together [14], [18]. Here it has been possible to reconstitute and partially characterize the two different types of N-P complexes (Oligomer N-P, Monomer N-P) separately under *in vitro* conditions. Moreover, this data further substantiates the differential interactions of N and P proteins when expressed in live cells.

### N protein utilizes its N and C terminal domains independently for interaction with P in its monomer and oligomer form respectively

**Figure 4:**
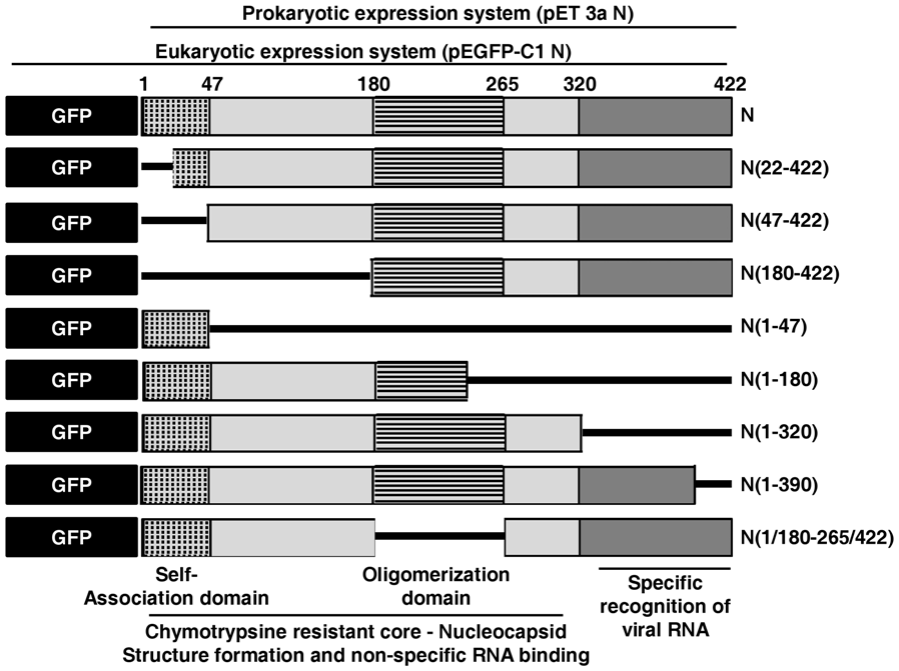
Schematic representation of the wild-type N protein and the mutant N proteins used in this study. Prokaryotic clones were made in pET3a vector for bacterial expression. Eukaryotic clones were made in pEGFP-C1 vector as N terminally GFP fused proteins. Different functionally relevant domains are also shown [21]. The numbers represents amino acid positions.

Next, to establish the domain(s) of the N protein involved in its interaction with P, we have employed a set of deletion mutants of N previously described in reference [21]. Figure 4 shows a schematic representation of these deletion mutants along with the functional domains involved in self-association and RNA binding [21]. However, for this study we have included two additional deletion mutants, N(22–422) and N(1–390). According to the crystal structure of VSV N, N-terminal 22 amino acids constitute an extended arm which interacts with the C-lobe of the preceding N molecule [32], [40]. However, upon deletion of these amino acids, the truncated N still conserves its ability of self-association (albeit with lower stability) and its association with P, as described previously [40]. We therefore included N(22–422) in our studies to check if this phenomenon is also true for CHPV N. Also, the crystal structure of VSV N-RNA-P complex suggests that first 390 amino acids retains the C-terminal P interacting site of N, and is therefore sufficient for its interaction with P [32]. However, according to Takacs et al., the extreme C-terminal end of VSV N plays a crucial role in VSV N-P interaction [41]. We have therefore included N(1–390) in the following studies to evaluate the importance of the C-terminal 32 aa in CHPV N-P interaction. All these mutants were expressed in BL21(DE3) or in BL21(DE3)pLys S and purified by anion exchange chromatography (MonoQ 5/50 GL) as described under materials and methods and in [21]. An N-terminal six histidine tagged variety of the P protein (His-P) was used for studying N-P interaction *in vitro*. The purified truncated proteins were subjected to size-exclusion chromatography through S-200 column to ensure their oligomerization status as was reported previously [21]. The two new mutants N(1–390) and N(22–422) were found to retain their propensity to form oligomeric structures like wild-type N (Table 1).

**Table 1:** Comparison of oligomerization status and cellular distribution of different truncated mutants of CHPV N.

Truncated Proteins	Oligomerization status	Cellular Distribution
N(22–422)	Octamer	Punctate
N(48–422)	Monomer	Homogeneous
N(180–422)	Monomer	Homogeneous
N(1–47)	Trimer	Homogeneous
N(1–220)	Tetramer	Punctate
N(1–320)	Octamer	Punctate
N(1–390)	Octamer	Punctate
N(1/180–265/422)	Trimer	Punctate

Oligomerization status was estimated by size-exclusion chromatography described previously (21), and cellular distribution was assessed by expressing GFP fused constructs in Vero-76 cells, followed by confocal fluorescence microscope.

**Figure 5:**
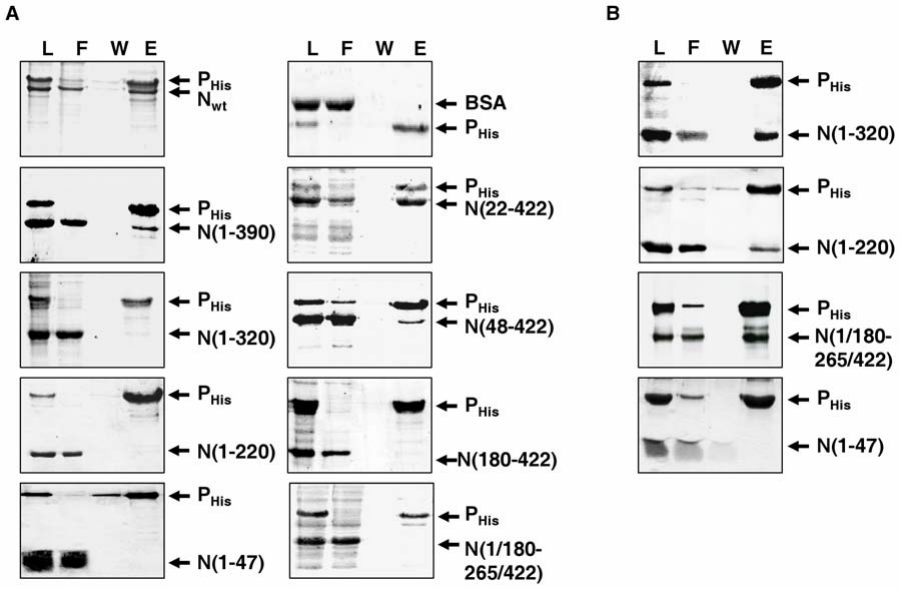
N protein utilizes two separate domains for interacting with P in its monomeric and oligomeric forms. N-terminally His-tagged P protein (His-P) was allowed to interact with either wild-type N or different N mutants in 100 mM NaCl TET buffer containing 10 mM Imidazole for 30 minutes at 4°C. Reaction mixtures were applied to Ni-NTA column and elution profile assayed by silver staining. L- loading; F- flow through; W- 10 mM Imidazole wash; E- 250 mM Imidazole elution. (A) In the absence of 1% DOC treatment. Bovine Serum Albumin (BSA) was used as negative control. (B) Wild-type N or N mutants were pre-incubated with 1% DOC for 30 minutes, followed by dialysis in presence of His-P, before applying to Ni-NTA column. Samples were resolved in 12% SDS-PAGE and visualised by Coomasie brilliant blue staining.

Next, His-tag co-elution assay was employed to determine the interacting ability of these truncated N proteins with the full length His-P. To this end, His-P was incubated either with wild-type or different truncated N proteins and the resulting mixture was then subjected to binding with Ni-NTA resin and subsequently eluted as described in material methods (Figure 5A). Co-elution of wild-type N with His-P confirms its ability to interact with P in its oligomeric form. It should be remembered that this interaction represents binding of P with nucleocapsid like structures of N [21]. To authenticate the specificity of this interaction, BSA was included which failed to co-elute with P. Deletion from the C-terminal end of CHPV N was found to weaken this interaction significantly, as a considerable amount of N(1–390) was observed in the flow-through. However, a significant fraction also co-eluted with P (Figure 5A). Further deletion from the C-terminal end completely abrogated the P interaction ability and N(1–320), N(1–220), N(1–47) failed to co-elute with P completely. Together, these data indicate the presence of a P interacting domain at the C-terminal end of CHPV N, residing within 320–390 amino acids. However, further stabilization through residues 390–422 cannot be ruled out from this observation. Interestingly, the N-terminal deletion mutant N(180–422) or the middle deletant N(1/180–265/422); although retains their C-terminal domain intact, failed to interact with P. In this context it should be noted that these mutants are oligomerization defective and exists in monomer and dimer forms respectively (Table 1). It is therefore evident that in CHPV, the C-terminal P interacting domain of N is highly dependent upon the oligomerization status of the protein, and can only be active in the characteristic nucleocapsid like structure of N. This claim is substantiated by the fact that the N-terminal deletant, N(22–422), which can form oligomeric structures like wild-type N (Table 1) and also retains its C-terminal domain intact, can interact with P. This observation is in agreement with the crystal structure of VSV N-P_CTD_ complex which demonstrated the presence of such C-terminal P binding domain active only in the ring shaped decameric structures of the protein [32]. Strikingly, the co-elution ability of the mutant N(48–422), which exists exclusively in the monomer form, points towards the existence of another P interacting domain active in the monomeric form of the protein. Further, inability of the monomer N(180–422) to interact with P, indicates that this P interacting domain is probably located at the N-terminal end of the protein.

To verify this possibility, we have slightly modified our His-tag co-elution experiment. As described in Figure 3, P can interact with 1% DOC treated monomeric N and form N^0^-P complex after removal of DOC. This phenomenon was used to verify the ability of the C-terminal deletion mutants N(1–320), N(1–220) and the middle deletant N(1/180–265/422) to interact with His-P protein in their monomeric form. It is noteworthy that, although these mutants retain the proposed N-terminal P binding domain, they failed to interact with P protein in their native self-associated form (Figure 5A). Hence, these mutants were treated with 1% DOC to dissociated there self-associated structures and then subsequently dialysed in presence of His-P protein. With the dialysed mixture co-elution assay was carried out as described above. Interestingly, prior incubation with DOC resulted in co-elution of all the three mutants with His-P protein (Figure 5B). These data together confirms that the N protein has an additional P interacting domain(s) which is only accessible in the monomer form of the protein. The ability of N(1/180–265/422) to interact with P narrows down this interacting region within the N-terminal 180 amino acids of N. However, it is worthwhile to note that mutant N(1–220) has a much weaker co-elution ability than N(1–320) or N(1/180–265/422). This suggests that the residues 265–320 of N (which are present in N(1/180–265/422) and N(1–320), but not in N(1–220)) may also have a role in binding P under monomeric conditions. In an attempt to further characterise the interacting region, we also checked the P interaction ability of DOC treated N(1–47). Failure of this mutant to interact with P suggests that the first 47 amino acids of CHPV N may not play an important role in N^0^-P interaction. However, it must be considered that due to its small size this mutant may not fold correctly into its functionally relevant form.

### Deletion of N-terminal 180 amino acids of N is sufficient to abrogate N-P interaction in cells

**Figure 6:**
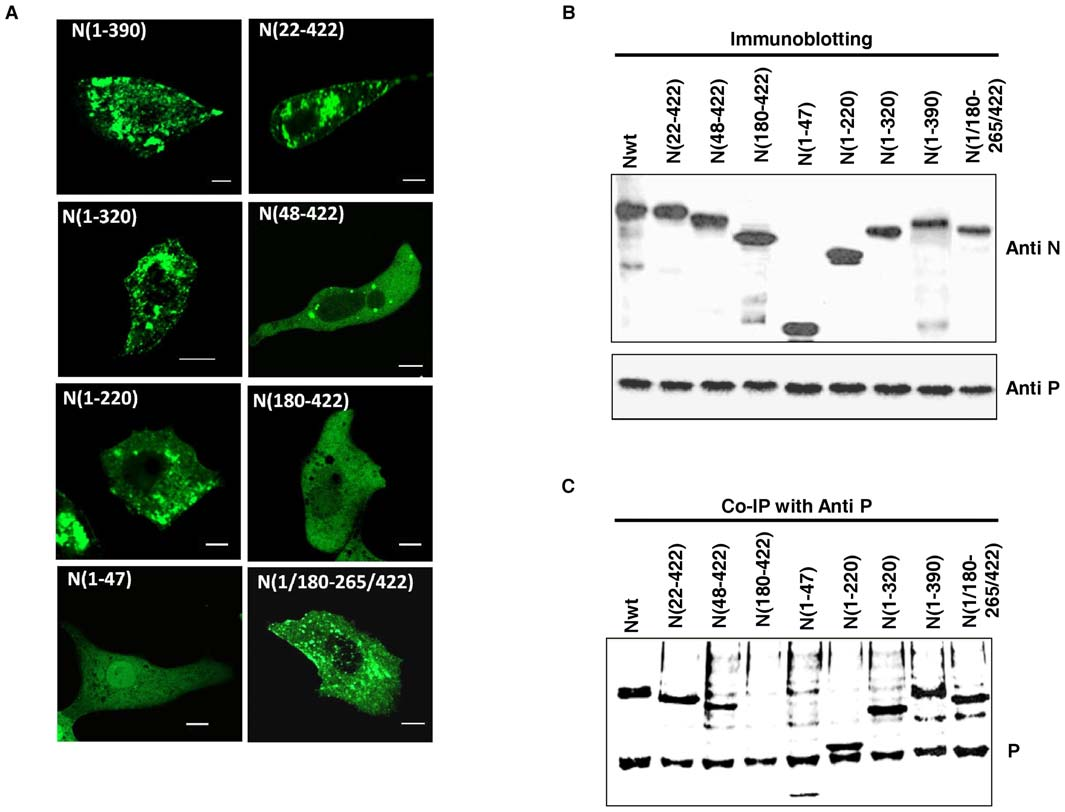
*Ex vivo* expression and immunoprecipitation of different N mutants with P. (A) Intra-cellular distribution of different mutants of N used in this study. Vero-76 cells were transfected with 2 µg of pEGFP-C1 constructs of each mutant. N-terminal deletants N(48–422) and N(180–422) exhibits smooth distribution. N(1–47) also exhibits smooth distribution, probably because of the large GFP-tag, which interferes with its oligomerization. The bar represents 5 µm. (B) Co-expression of wild-type N and different N mutants with P protein in Vero-76 cells. Co-expression was confirmed by immunobloting with N and P Abs (upper and lower panels, respectively). All of the mutants used for this study expresses satisfactorily, and is of the right relative size. (C) Co-immunoprecipitation of wild-type N and different N mutants with P protein. Vero-76 cells were co-transfected with 2 µg of both plasmids, labelled with L-Methionine-^35^S 24 hours post-transfection followed by immunoprecipitation with P Ab. Except for N(180–422), all mutants co-immunoprecipitate with P.

Following the lead from our *in vitro* his-tag co-elution assays which indicated the presence of two independent P interacting domains in N, we decided to further validate the result by co-immunoprecipitation experiment in transfected cells. The different truncated mutants of N were checked for their P interacting ability in the cellular milieu. All of the above mentioned mutants were cloned into eukaryotic expression vector pEGFPC1 with a GFP tag at its N terminal end (Figure 4). Mutants were expressed in Vero-76 cell line and expression was confirmed by immunoblotting with N-Ab. Electrophoretic pattern of all the mutants were in agreement with their expected molecular weight. It must be mentioned that, intracellular distribution pattern of these truncated N proteins, as monitored through confocal microscopy (Figure 6A), could be correlated with their oligomerization status as tabulated in Table 1. All three deletion mutants N(1–390), N(1–320), N(22–422), confirms their oligomerization propensity by formation of cytoplasmic punctate structures. The mutants existing as monomers, i.e. N(48–422) and N(180–422) demonstrated homogeneous distribution throughout the cytoplasm. Interestingly, the two mutants having defective oligomerization tendency, N(1/180–265/422) or N(1–220) (Table 1), also showed formation of punctate structures along with a homogeneous distribution in the background. Probably, such distribution represents a mixed population containing soluble and aggregated forms of these mutants in the cellular milieu. *In vitro*, N(1–47) forms trimers along with higher oligomers ([21] and Table 1). However, it exhibited a homogeneous distribution pattern when expressed as a GFP fused protein. Probably, GFP has diverse effect upon the self-association ability of this short peptide and is responsible for its soluble homogeneous distribution. Taken together, it is evident that the punctate distribution of wild-type or truncated N proteins in transfected cells is a manifestation of their self-association tendency.

Next, we tested the P interacting ability of the GFP-tagged wild-type N and truncated N mutants when co-expressed in Vero-76 cells. 24 hrs post transfection, proteins were metabolically labelled with L-Methionine-S^35^ and co-expression confirmed by immunoblotting with N Ab and P Ab (Figure 6B upper and lower panel). Interaction was monitored by the ability to co-precipitate with the P protein using P Ab (Figure 6C). GFP-N was used as a positive control. In this context, it should be noted that during co-expression, both monomer and oligomer forms of N are available for interaction with P protein. All but one of the mutants was found to be able to co-precipitate with P protein. Clearly, mutants that can form proper oligomeric structures and retains their C-terminal P-interacting region (320–390aa) intact, can bind P, i.e. N(22–422) and N(1–390). This reinforces the involvement of 320–390 aa of N in oligomer N-P interaction. On the other hand, mutants either lacking this C-terminal region i.e. N(1–320), N(1–220) or unable to form characteristic oligomeric structures i.e. N(48–422), N(1/180–265/422) are also found to interact with P. This can only be explained in light of the N-terminal P interacting region of monomeric N. Only N(180–422) failed to interact with P, as it neither has an intact N-terminal P interacting domain, nor can it form proper oligomeric structures that is necessary for the utilization of the C-terminal domain for binding P. N(1–47) showed P interaction ability under this condition, albeit to a lesser degree than other mutants. Taken together, this co-immunoprecipitation data strongly supports our *in vitro* His-tag co-elution results, confirming the presence of two independent P binding domains within N.

### P inhibits oligomerization of N by partially masking its self-association domain

From the data presented so far, it is evident that in addition to the C-terminal P binding domain, the CHPV N possesses another unique N-terminal P interacting region which is functional only in the monomeric form of the protein. Now, the self-association domain of N has been reported to reside in the chymotrypsin resistant N-terminal 320 amino acids of the protein [21]. On the basis of these two observations we hypothesize that P partially masks the self-association domain of N by interacting with residues vital for N-N self association and thereby maintains N in its monomeric form (N^0^).

**Figure 7:**
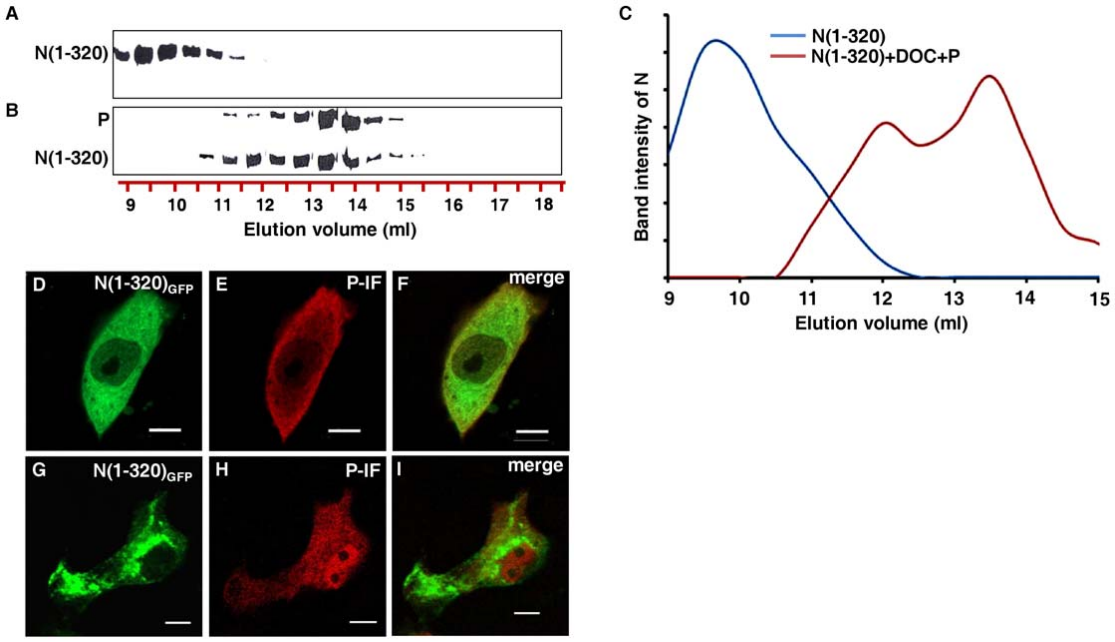
Monomers of N(1–320) binds to P, but oligomers do not. Size exclusion chromatography of bacterially expressed purified N(1–320) and P proteins, visualised by Coomasie brilliant blue staining. (A) N(1–320) alone. (B) N(1–320) treated with 1% DOC (to dissociate the oligomers into monomers) for 30 minutes and subsequently dialysed to remove DOC in the presence of equimolar concentrations of P protein. Co-elution of the two proteins confirms that N(1–320) can bind to P in monomeric state. (C) Plot representing densitometric scans of N(1–320) bands with respect to elution volume in ml. (D) GFP fluorescence of Vero-76 cells co-transfected with 1∶1 ratio of pEGFP-C1 N(1–320) and pCDNA 3.1 (+) P. Smooth distribution of N(1–320) is observed. (E) Immunofluorescence of the cells in D with P Ab. P also shows smooth distribution. (F) Merge of D and E. (G) GFP fluorescence of Vero-76 cells co-transfected with 1∶0.5 ratio of pEGFP-C1 N(1–320) and pCDNA 3.1 (+) P. N(1–320) shows punctuated distribution. (H) Immunofluorescence of the cells in G with P Ab. Distribution of P is smooth. (I) Merge of G and H. All data were captured on a laser scanning confocal microscope (Carl Zeiss). P Immunofluorescence were performed with anti-rabbit TRITC conjugated secondary antibody. 2 µg of DNA was used for all transfection, except for G, H and I where 1 µg of pCDNA 3.1 (+) P was used. The bar represents 5 µm.

To validate our hypothesis, we have chosen the mutant N(1–320) which lacks the C-terminal P interacting region but possesses the N-terminal one. Also, this is the largest possible C-terminal deletion of the protein retaining the ability to form characteristic oligomeric structures like the wild-type N [21]. Now, if our hypothesis is true, P should be able to exhibit chaperone like activity upon this mutant and therefore inhibit its self-association. To evaluate this, we dissociated the oligomers of N(1–320) with DOC and then allowed them to re-associate by dialysing out the DOC in presence or absence of P. Similar experiment with wild-type N resulted in formation of monomer N-P complex at the expense of oligomers (Figure 3). Interestingly, oligomerization of N(1–320) was also significantly inhibited in presence of P, as evident from the gel-filtration profiles (Figure 7A, B and C). In contrast to DOC untreated N(1–320), which eluted close to the void volume of the S-200 column at 9.5 ml, DOC treated N(1–320) formed a number of low molecular weight complexes with P, majority of which eluted at 13.5 ml (Figure 7A, B and C). Estimated molecular weight of this complex (∼70 kDa) is in the close proximity to that of a heterodimer composed of one N(1–320)^0^ and one P. However, complexes of other stoichiometry are also observed. This certainly suggests that a major fraction of N(1–320) is retained in its monomer form by forming a 1∶1 complex with P. DOC treatment does not have any permanent effect upon the oligomerization ability of the protein as was evident from the identical gel-filtration profile of the DOC removed and untreated sample (not shown). In agreement with the His-tag co-elution data, P fails to interact with oligomeric N(1–320) (DOC untreated) and therefore, does not have any effect upon its gel-filtration profile . Thus, the N-terminal 320 amino acids of CHPV N have all the necessary contacts to interact with P under monomeric conditions.

Next, to substantiate this observation *ex-vivo*, we monitored the effect of co-expression of P upon the cellular distribution of GFP-N(1–320). As evident from Figure 7D, co-expression of P in 1∶1 ratio significantly affect the aggregation tendency of N(1–320) and resulted in a smooth distribution of this protein throughout the cytoplasm (compare Figure 6A, N(1–320) and 7D). Co-expression of P and its co-localization with N were verified by immunofluorescence with P Ab (Figure 7E and F). Further, co-expression of N(1–320) with P in 1∶0.5 ratio did not have any such effect upon the cellular distribution of the aggregated structure of N(1–320) (Figure 7G), as was the case for wild-type N. However, interestingly in this case, unlike the interaction of P with wild-type N under similar conditions, here, P failed to co-localize with the aggregated structures of N as evident from the immunofluorescence data (compare Figure 7G, H, I with Figure 1H, I, J). Clearly, P could not interact with the oligomers of N(1–320) due to the absence of the C-terminal domain of N, as also evident from the *in vitro* gel-filtration analysis.

## Discussion

Recent work with the CHPV N protein has put an emphasis on the obligatory role of monomer N (N^0^) in specific encapsidation of the viral genome in presence of large excess of cellular RNAs, during viral replication [21], [29]. However, maintenance of N in an encapsidation competent monomer form is entirely dependent upon its interaction with the P protein, i.e. the formation of a N^0^-P complex [26], [28], [29]. Regions of VSV or RAV P protein involved in this interaction have been found to be situated at the extreme N-terminal end of the protein [14], [37], which is distinct from the nucleocapsid binding C-terminal domain (P_CTD_) [32]. Reports with Sendai virus also supported the presence of such N^0^ binding domain at the N-terminal region of P [35]. However, for the N protein, a major void has remained in regard to the regions of N^0^ that participate in this interaction. This is primarily due to the lack of soluble preparations of monomeric N (N^0^), which till date has been a major hurdle to structural and biochemical study of the N^0^-P complex.

Co-expression of VSV N and P proteins in different expression systems results in formation of multiple N-P complexes of various stoichiometries [24]. Among them, two complexes have been found to be functionally relevant during the viral infection cycle, namely, a 2∶1 N-P complex corresponding to the decamer-N-RNA-P interaction, and a complex representing the interaction of monomer N with monomers or dimers of P (N^0^-P). It appears that, within the system, these two complexes remain in equilibrium, and probably for this reason, it is difficult to modulate the abundance of one of these complexes at the expense of the other. Here we have been able to reconstitute the formation of both oligomer N-P and monomer N-P complexes *in vitro*, independent of one another. Incubation of individually expressed (bacterial) soluble N and P proteins together, resulted in a high molecular weight complex, composed of octameric/decameric nucleocapsid like structures of N associated with P in 2∶1 ratio. This complex vastly resembles the VSV N-P complex encountered by Green et al. while co-expressing N-P in *E. coli* [18]. It is representative of the interaction of P as a polymerase co-factor with the nucleocapsid template [32]. It is thus noteworthy that, P binding ability is hardwired into the nucleocapsid like structures of N and is retained even if expressed independently. In contrast, reversible disruption of the oligomers of N with the dissociating detergent, Sodium Deoxycholate (DOC) resulted in generation of N^0^ [29], which was also found to interact with P (Figure 3) to form N^0^-P complex. Moreover, formation of this complex resulted in inhibition of N-N self-association, re-establishing the role of P in retaining N in its monomeric form. Interestingly, these *in vitro* results are in accordance with our *ex-vivo* data, where dual mode of N-P interaction is reflected when co-expressed in live cells. According to our observations, co-transfection of N and P in a 1∶1 ratio resulted in solubilisation of aggregated N, and co-localization of the two proteins together, indicating N^0^-P complex formation. However, co-transfection in a 1∶0.5 ratio resulted in co-localization of the otherwise homogenous P, with punctate N, which we deem to be representative of oligomer N-P complexes. A similar observation has been described by Omi-Furutani et al. for the Nipah virus [42]. Therefore, it is evident that by varying the availability of P protein we can alter the mode of N-P interaction in live cells.

In the present work, we have successfully identified two unique P interacting regions in the CHPV N protein. While a C-terminal region has been found to bind P only under oligomeric conditions, another N-terminal region has been identified, which mediates interaction of monomeric N with P (N^0^-P). Primary indications for the possibility of these two independent domains came from His-tag co-elution assays with different truncated versions of the N protein, either untreated or pre-treated with 1% DOC. It has been found that DOC untreated oligomer N possesses a C-terminal P binding domain residing between residues 320–390. However, this P binding domain is only active in the proper nucleocapsid like decameric structures of N, as evident from the incapability of mutants N(180–422) and N(1/180–265/422), to interact with P under similar conditions. These mutants although retain their C-terminal domain intact, lacks the ability to form proper nucleocapsid like structures like the wild-type protein. Presence of such P binding site in the nucleocapsid has also been observed previously by the crystal structure analysis of VSV nucleocapsid-RNA-P complex [32]. According to this report, in VSV N, a contiguous stretch of residues (354–386) in the C-terminal domain of P, including helix α-13 and the extended loop of the C lobe, form the P interacting site. Moreover, in decamers of N, this P binding domain from two neighbouring N monomers come together to form a unique P binding site, thereby restricting such P binding activity to the nucleocapsid structure only. This could be a general strategy for both VSV and CHPV to minimize the chances of recruitment of viral polymerase to defective nucleocapsid structures, thereby optimizing their RNA synthesis. Also, possibilities of additional residues that may play role in this interaction could not be excluded. This is because, in the case of N(1–390), P interaction ability is significantly affected by deletion of the extreme C-terminal 32 amino acids. This result that can be explained by considering the observation made by Takacs et al. [41] which suggested the involvement of C-terminal 5 amino acids in N-P interaction. It seems that although the actual P binding site resides within 320–390 amino acids of N, the extreme C-terminal region is also involved in maintaining proper conformation of the P binding site.

On the other hand, P interacting ability of N(48–422) in the His-tag co-elution assay, opens up the possibility of a new P interacting region within N. This is because, N(48–422) is incapable of oligomerization and exists solely as a monomer, and thus, does not have a functional P_CTD_ interacting site. This possibility was further validated by P interaction ability of the mutants N(1–320), N(1–220), N(1/180–265/422) in their monomer forms generated by prior treatment with DOC. N(1/180–265/422) is interesting in this respect because, though it does not form decameric structures, it is capable of forming dimers (Table 1), which, as evident from the data presented here is not adequate to bind to P. Therefore, this N-terminal P binding domain is only functional in the monomeric form of the protein. The ability of N(1/180–265/422) to interact with P upon DOC treatment reduced this binding domain to the first 180 residues of the N protein. However, the decreased binding of N(1–220) as compared to N(1–320) and N(1/180–265/422) under DOC treated conditions, also indicates the possible role of residues 265 to 320 in this interaction. This His-tag co-elution data was further supported by co-immunoprecipitation assays where all of the mutants, except N(180–422), co-precipitated with P, when the two proteins were co-expressed in Vero-76 cells. Therefore, it is again evident that the first 179 amino acids of monomer N are indispensible in binding P. Interestingly, N(1–47) which failed to interact with P in DOC treated or untreated forms in the His-tag co-elution assay, co-precipitated with P when co-expressed. In this context, it is noteworthy that this short peptide has high oligomerization tendency when expressed in *E. coli* but loses the same upon expression as a GFP-fusion protein. Probably the GFP fusion affects the structure of this short peptide and is responsible for its anomalous behaviour.

A challenging question that remains is how interaction with P inhibits the self-association of N and therefore, maintains it in its soluble form. We have previously shown that formation of helical nucleocapsid like structure of CHPV N is dependent upon the interaction of the N-terminal arm (residues 1–47) of one N monomer with the central region (residues 180–265) of another adjacent N moiety [21]. Any interference in these sites of interaction should either result in total abrogation of oligomer formation or in defective self-association. Interestingly, our data indicates that the N-terminal P interacting region of N partially coincides with its oligomerization domain. We therefore propose that association of P to this N-terminal binding site of nascent N either partially or completely masks the self association domains of N, resulting in maintenance of N in its monomeric form (N^0^). Support in favour of this mechanism came when DOC treated N(1–320) was allowed to form oligomers in presence of the P protein. It must be remembered that N(1–320) is the smallest possible C-terminal truncation of the N protein retaining its ability to form characteristic oligomeric structures like the wild-type protein [21]. Gel-filtration analysis clearly indicates that presence of P severely affects the self-association tendency of DOC treated monomer N(1–320), which co-elutes as a slower migrating N(1–320)^0^-P complex. Furthermore, co-expression of N(1–320) with P in live cells in 1∶1 ratio resulted in redistribution of the punctate structures of this protein into a homogeneous form, confirming the chaperone like activity of P upon N(1–320). Together, these data confirms that interaction of P with the N-terminal portion of N is sufficient to block the N-N self-association and subsequently inhibit the oligomerization process. Our data validates the prediction made by Curran et al. [35] where it had been proposed that interaction of P at the self-association domain of monomer N prevents N^0^-P from aggregation. However, in a pre-formed nucleocapsid this domain is involved in interaction with neighbouring N subunits and hence unavailable for interaction with P. In such form, the C-terminal domain represents the only binding site for P. A similar observation has been reported recently by Leyrat et al., where they have shown that the N^0^-binding region of P competes with the N terminal arm of a neighbouring N molecule, thus preventing N-N self assembly [33], [43].

**Figure 8:**
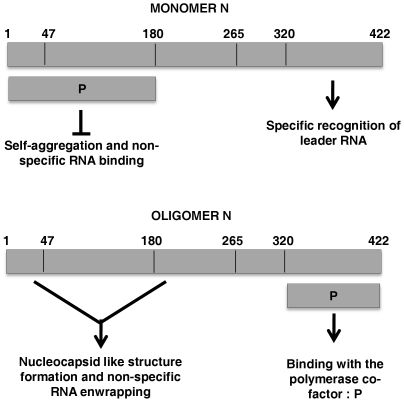
Schematic representation of the domains of CHPV N involved in interaction with P and their functional importance. Binding of P to nascent N masks the N-N self association region of CHPV N (N^0^-P complex formation) and also blocks non-specific RNA binding (upper panel). N^0^ is capable of specifically recognizing the viral leader sequence and the C-terminal 102 amino acids are essential for this recognition. Therefore, in the monomeric form, N specifically recognizes the leader RNA, to form the nucleation complex. Subsequently, the process of N-N self-association begins and P is released. Upon oligomerization, a new RNA binding cavity is formed utilizing the N-terminal arm (1–47 aa) and the central region of N (lower panel). Thus, the phase of non-specific encapsidation begins. Once nucleocapsids have formed, P can again interact with N, this time with the C-terminal region of oligomeric N, to usher the viral polymerase (L) onto its template.

Finally, we present a model (Figure 8) that explains the specific encapsidation of viral RNA during viral genome replication. Nascent N is maintained in an encapsidation competent soluble form (N^0^) by its interaction with the P protein (by N^0^-P complex formation). In this context, P masks the self-association domain of N and thus maintains it in a monomer form. Previously, we have shown that CHPV N^0^ is capable of specifically recognizing the viral leader sequence and the C-terminal 102 amino acids are essential for this recognition. On the other hand, upon oligomerization, a new RNA binding cavity is formed utilizing the N-terminal arm (1–47 aa) and the central region of N [21]. Therefore, the P bound monomeric N specifically recognizes the leader region of the viral genome RNA, to form the nucleation complex. This may be a transient state, immediate to which, the process of N-N self-association begins. For this purpose, the P has to be released by a yet unknown mechanism. The polymerase L may have a role in this process. Studies with VSV indicated that P shares an overlapping N-terminal region for interaction with L [44] and with N^0^ [14]. Hence, interaction with L, within the close proximity of the N^0^ binding site could be a thrust to replace the N from P. Release of P from N^0^ unmask its self-association domain, hence can trigger the N-N self-association to begin, leading to helical nucleocapsids. During this self-association, a new RNA binding surface is generated using the N-terminal two-third of N, which is capable of accommodating diverse RNA sequences in the elongation phase of encapsidation [21]. In this context, it is worth mentioning that the N-terminal non-specific RNA binding domain is only available in self-associated N [21]. Once nucleocapsids have formed, P can again interact with N, this time with the C-terminal region of oligomeric N, to usher the viral polymerase (L) onto its template.

It is interesting to note that CHPV nucleocapsid not only shares common architecture with other viruses in the *Rhabdoviridae* family [21] but also employs common strategy for encapsidation of its genome RNA. This also point towards the possibility of existence of similar N-terminal P binding regions in the N proteins of other *Mononegalovirales* as well. This newly elucidated P binding region may serve as a potential target for designing novel therapeutics interventions.

## Materials and Methods

Oligonucleotides used in this study were purchased from Integrated DNA Technologies (IDT), USA. All column chromatography materials were from GE Healthcare. Ni-NTA resin was from Qiagen. Foetal Bovine Serum, antibiotics and Trypsin-EDTA needed for cell culture purpose were supplied by Invitrogen™ (GIBCO). Radioactive biomolecules were from BRIT, India. All other chemicals and biochemicals were of analytical grade.

### Cell lines

Vero-76 cell lines supplied by NCCS, Pune were grown as monolayers in DMEM enriched with 10% FBS, 100 U/ml Penicillin-Streptomycin, 2 mM Glutamine in tissue culture treated flask (BD Biosciences) in CO_2_ incubator at 5% CO_2_, 80% humidity and 37°C temperature.

### Construction of plasmids and expression of the wild-type and deletant proteins

Different GFP tagged, truncated versions of CHPV N were created by PCR amplification from pET3a-NC [21] and subcloning into pEGFP-C1 vector by using BamHI and KpnI restriction enzymes (Fermantas, Thermo scientific) (see Table 2 for primers used in this process). GFP tagged N(1/180–265/422) was obtained by a similar strategy using previously available pET3a-N(1/180–265/422) as the template [21]. N(48–422) and N(180–422) for bacterial expression was created by subcloning into pET3a using pET3a-NC as template. All clones were confirmed by sequencing.

**Table 2:** Oligonucleotides used for the construction of the GFP fused truncated N proteins.

Construct	Forward Primer (KpnI)	Reverse Primer (BamHI)
pEGFP-C1 N(22–422)	CGGGGTACCGACCCAGTGGAGTTTCCA	TTTATAGGATCCTCATGCAAAGAG
pEGFP-C1 N(48–422)	CGGGGTACCGATCTGAGTCTTTTGAGGAG	TTTATAGGATCCTCATGCAAAGAG
pEGFP-C1 N(180–422)	CGGGGTACCGAATTCTTCAATGCTTGGGC	TTTATAGGATCCTCATGCAAAGAG
pEGFP-C1 N(1–47)	CGGGGTACCATGGCCAGTTCTCAAGTATTCTGCATTT	CGCGGATCCTCATGTCTCCTTCTTTATGTACAC
pEGFP-C1 N(1–220)	CGGGGTACCATGGCCAGTTCTCAAGTATTCTGCATTT	CGCGGATCCTCACACAATTGTTCCGAAACG
pEGFP-C1 N(1–320)	CGGGGTACCATGGCCAGTTCTCAAGTATTCTGCATTT	TTTATAGGATCCTCATGGAACTAAAGCATTCTT
pEGFP-C1 N(1–390)	CGGGGTACCATGGCCAGTTCTCAAGTATTCTGCATTT	TTTATAGGATCCTCAAATTTCATGCTTAATATCCT
pEGFP-C1 N(1/180–265/422)	CGGGGTACCATGGCCAGTTCTCAAGTATTCTGCATTT	TTTATAGGATCCTCATGCAAAGAG

All full-length and deletion mutants of N were expressed in *E. coli*, BL21(DE3) or in BL21(DE3) pLysS. The expression and purification of full-length untagged protein was carried out as described earlier [21], [27]. Wild-type P (pET3a-P) and 6×His tagged P (pET20b-P) were purified by Q-Sepharose anion exchange resin (GE Healthcare) as described previously by Chattopadhyay et al. [45] or Ni-NTA agarose (Qiagen) according to manufacturers protocol. Eukaryotic expression of wild-type GFP-N (pEGFP-C1 N), Wild-type untagged P (pCDNA 3.1(+) P), has been described previously [16].

### Gene Transfection

One day prior to performing transient transfection, 35 mm tissue culture plates (containing coverslips for immunofluorescence) were seeded with 4×10^5^ Vero-76 cells per well. Cells were transiently transfected with 2 µg of either pEGFP-C1 containing cDNA of wild-type or mutant forms of CHPV N alone, or co-transfected with 2 µg (or 1 µg) pCDNA 3.1(+) containing cDNA of CHPV P. Transfection were performed with 6 µl of TurboFect™ *in vitro* Transfection Reagent (Fermentas) according to manufacturer's protocol.

### Immunofluorescence

Polyclonal anti-CHPV N and anti-CHPV P has been described previously [21], [46]. Immunofluorescence of cultured cell was performed according to standard protocol. Briefly, 24 hours post-transfection, cells were washed with ice cold phosphate buffer saline (PBS) twice, and fixed with 2% para-formaldehyde (PFA) at room temperature for 40 minutes. After thrice washes with PBS, cells were permeabilized with 10 mM Na-citrate, pH-6.0 containing 0.1% Triton X 100 at room temperature for 20 minutes and blocked with 5% bovine serum albumin in PBS (Invitrogen, USA) for 1 hour. Primary antibody (1∶200) was treated at 4°C, overnight. Anti rabbit secondary TRITC conjugated antibody was added (1∶300) after three PBS washes. After incubation for 1 hour, cells were washed three times with PBS. Finally, the coverslips were mounted with anti-fade mounting media and evaluated using the ×63 objective of a confocal microscope (Carl Zeiss) equipped for GFP visualization (488-nm excitation and FITC filter set) and TRITC visualization (543-nm excitation and TRITC filter set). The images were corrected for possible cross-talk by sequential scanning in multiple channels using the multi-track configuration.

### Soluble-insoluble fractionation and sucrose density gradient centrifugation

At 24 hrs of post-transfection, cells were washed with phosphate buffer saline (PBS) and lysed in lysis buffer (20 mM Tris-HCl (pH 8), 150 mM NaCl, 1 mM EDTA, 1 mM EGTA, 1% TritonX-100, 5 mM DTT) and sonicated on ice with a 5 second pulse. After centrifugation at 13000 rpm at 4°C for 30 minutes, the supernatant containing the soluble protein pool were layered over a 10–60% sucrose step gradient and were centrifuged for 16 hours at 32000 rpm in a SW44 Beckman rotor at 4°C. 0.5 ml fractions were collected from the bottom of the gradients, precipitated with Trichloroacetic acid (TCA) and resolved in a 12% sodium dodecyl sulphate-polyacrylamide gel electrophoresis (SDS-PAGE). Gels were Western blotted (WB) with N and P antibodies to check their distribution pattern, according to standard protocols. Densitometric analysis of WB were performed with ImageQuant™ TL software (GE Healthcare) within the linear dynamic range of detection.

### In vitro N-P interaction

Equimolar amounts of bacterially expressed, purified N and P proteins were incubated together in 100 mM NaCl containing TET buffer (50 mM Tris pH 8.0, 1 mM EDTA and 0.1% Triton X-100), at 4°C for 30 minutes. The resulting complex was then subjected to gel-filtration chromatography through S-200 (10/300) column (GE Healthcare), pre-equilibrated in the same buffer. For dissociation of the N oligomers, N was pre-treated with 1% Sodium Deoxycholate (DOC) at room temperature for 30 minutes followed by overnight dialysis against 100 mM NaCl containing TET buffer in presence of equimolar amounts of P protein. The resulting adduct were then subjected to gel-filtration chromatography as mentioned above. 0.5 ml fractions were collected, and resolved on 12% SDS-PAGE. Bands were visualised by silver staining and densitometric analysis of bands were performed with ImageQuant™ TL software (GE Healthcare) within the linear dynamic range of detection.

### His-tag co-elution assay

Wild-type or truncated N proteins were incubated with 6×His-P protein in binding buffer (50 mM Tris-Cl, pH-8, 100 mM NaCl, 0.1% TritonX-100 and 10 mM Imidazole), at 4°C for 30 minutes and the resulting complexes were allowed to bind to Ni–NTA agarose pre-equilibrated in the same buffer. A 10 µl aliquot of the complex was kept aside before addition of Ni-NTA, as loading sample (L). Binding was allowed for 1 hour, and the flow-though (FT) was collected. After consecutive washings with 10 mM (W), 20 mM and 50 mM Imidazole, the proteins were finally eluted with 250 mM Imidazole (E). L, FT, W and E were resolved in 12% SDS-PAGE and visualised by Coomassie Blue Staining. Alternatively, N variants were treated with 1% DOC and dialysed against the binding buffer in presence of P protein. The complexes formed were subjected to the co-elution assay mentioned above.

### Co-immunoprecipitation

pCDNA 3.1-N and pCDNA 3.1-P were co-transfected into Vero-76 cells, grown in 35 mm culture plates. At 24 hours of post-transfection, cells were starved in DMEM lacking methionine and cysteine (PAN Biotech) for another 30 min and then exposed to 50 µCi/ml of ^35^S labelled methionine and cysteine (^35^S INVIVO ProTwinlabel; BRIT, India) in the same medium, for 2 hrs. Cells were then washed with PBS and lysed as mentioned previously. Lysates were incubated overnight with polyclonal anti-P antibody (1∶400 dilution) and the protein complexes were immunoprecipitated using Protein-A Sepharose™ CL-4B (GE Healthcare) for one hour, according to manufacturer's instructions. After subsequent washing with lysis buffer, the sepharose beads were boiled with 1× protein loading dye for 5 minutes and samples were resolved in a 10% SDS-PAGE and subjected to autoradiography.

## Supporting Information

Figure S1 **CHPV P proteins acts as an N-specific chaperone when transfected at a 1∶1 ratio in Vero-76 cell lines.** Upon transfection of Vero-76 cells with GFP-N and P encoding constructs in a 1∶1 ratio, about 90% cells exhibited homogenization of the otherwise punctated distribution of GFP-N. The remaining 10% cells, were found to be lacking in the expression of P, and therefore showed characteristic punctated structures of N. This image shows the three possible types of cells in one field. Cells co-transfected with both GFP-N and P plasmids resulted in homogenization of N aggregates (N_GFP_+P). Cells that were transfected with GFP-N alone continued to exhibit punctated pattern of GFP-N distribution (N_GFP_), while cells that received P alone, showed typical homogenous distribution of P throughout the cytoplasm (P-IF). Immunofluorescence against P was performed with P Ab and anti-rabbit TRITC conjugated secondary antibody. Images were captured on a laser scanning confocal microscope (Carl Zeiss). The bar represents 5 µm.
(DOC)
