# Supplementary material for: Interaction of Chandipura Virus N and P Proteins: Identification of Two Mutually Exclusive Domains of N Involved in Interaction with P
Source: PLoS One. 2012 Apr 2;7(4):e34623. doi: 10.1371/journal.pone.0034623 (PMC3317646; doi:10.1371/journal.pone.0034623)
Supplement: Figure S1 — CHPV P proteins acts as an N-specific chaperone when transfected at a 1∶1 ratio in Vero-76 cell lines. Upon transfection of Vero-76 cells with GFP-N and P encoding constructs in a 1∶1 ratio, about 90% cells exhibited homogenization of the otherwise punctated distribution of GFP-N. The remaining 10% cells, were found to be lacking in the expression of P, and therefore showed characteristic punctated structures of N. This image shows the three possible types of cells in one field. Cells co-transfected with both GFP-N and P plasmids resulted in homogenization of N aggregates (NGFP+P). Cells that were transfected with GFP-N alone continued to exhibit punctated pattern of GFP-N distribution (NGFP), while cells that received P alone, showed typical homogenous distribution of P throughout the cytoplasm (P-IF). Immunofluorescence against P was performed with P Ab and anti-rabbit TRITC conjugated secondary antibody. Images were captured on a laser scanning confocal microscope (Carl Zeiss). The bar represents 5 µm. (DOC) [file pone.0034623.s001.doc]

**Supporting Information**

Figure S1


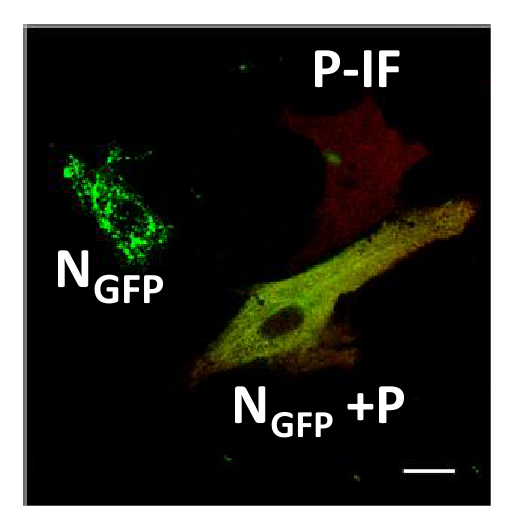


**Figure S1: CHPV P proteins acts as an N-specific chaperone when transfected at a 1:1 ratio in Vero-76 cell lines.** Upon transfection of Vero-76 cells with GFP-N and P encoding constructs in a 1:1 ratio, about 90% cells exhibited homogenization of the otherwise punctated distribution of GFP-N. The remaining 10% cells, were found to be lacking in the expression of P, and therefore showed characteristic punctated structures of N. This image shows the three possible types of cells in one field. Cells co-transfected with both GFP-N and P plasmids resulted in homogenization of N aggregates (N_GFP_+P). Cells that were transfected with GFP-N alone continued to exhibit punctated pattern of GFP-N distribution (N_GFP_), while cells that received P alone, showed typical homogenous distribution of P throughout the cytoplasm (P-IF). Immunofluorescence against P was performed with P Ab and anti-rabbit TRITC conjugated secondary antibody. Images were captured on a laser scanning confocal microscope (Carl Zeiss). The bar represents 5 µm.
